# Does traffic‐related air pollution exposure alter blood gas parameters in recreationally trained male cyclists during prolonged endurance exercise?

**DOI:** 10.14814/phy2.70408

**Published:** 2025-06-01

**Authors:** André C. Silveira, Júlio S. Hasegawa, Ramon Cruz, Monique Matsuda, Mônica V. Marquezini, Adriano Eduardo Lima‐Silva, Paulo Saldiva, Michael S. Koehle, Rômulo Bertuzzi

**Affiliations:** ^1^ Endurance Performance Research Group (GEDAE‐USP), School of Physical Education and Sport University of São Paulo São Paulo São Paulo Brazil; ^2^ Unit of Environmental Condition and Endurance Performance Analysis (UACAMDA), Sports Center, Department of Physical Education Federal University of Santa Catarina Florianopolis Santa Catarina Brazil; ^3^ Laboratory of Investigation in Ophthalmology (LIM‐33), Division of Ophthalmology, Faculty of Medicine University of São Paulo São Paulo São Paulo Brazil; ^4^ Pro ‐ Sangue Foundation, São Paulo and Pathology Department, Faculty of Medicine University of São Paulo São Paulo São Paulo Brazil; ^5^ Human Performance Research Group, Academic Department of Physical Education (DAEFI) Federal University of Technology Parana Curitiba Parana Brazil; ^6^ Institute of Advanced Studies University of São Paulo São Paulo São Paulo Brazil; ^7^ School of Kinesiology University of British Columbia Vancouver British Columbia Canada

**Keywords:** cycling, exercise, gasometry, health, particulate matter

## Abstract

Exposure to air pollution has been a significant challenge in large cities as São Paulo, Brazil, particularly for individuals exercising outdoors. The increasing on ventilation (VE) during physical effort can lead to greater pollutant inhalation. Our goal in the present study evaluated whether air pollution exposure affects venous blood gases and if it has an impact on performance during a 50‐km cycling time trial (TT). Ten male cyclists performed the TT in an environmental chamber under TRAP and filtered air conditions. Venous blood samples collected pre‐ and post‐TT were analyzed for pH, PvCO_2_ (partial pressure of carbon dioxide in venous blood), PvO_2_ (partial pressure of oxygen in venous blood) hematocrit (Htc), hemoglobin (Hb), and oxygen saturation (SvO_2_). PM_2.5_ levels were significantly lower in filtered air (11.2 ± 4.7 μm/m^3^) than in TRAP (34.6 ± 10.8 μm/m^3^). There was no significant difference in mean power output between conditions (*p* = 0.907, *d* = 0.038). Blood gas parameters showed no condition effect or interaction, but time significantly affected PvO_2_ (*p* = 0.04), Hb (*p* < 0.01), Htc (*p* < 0.01), and PvCO_2_ (*p* = 0.02). These findings suggest recreationally trained cyclists experience no performance impairment under TRAP, with minimal changes in venous blood gas parameters.

## Graphical abstract

Changes in blood gas parameters following a 50‐km cycling time trial performed in a real‐world air‐polluted environment.

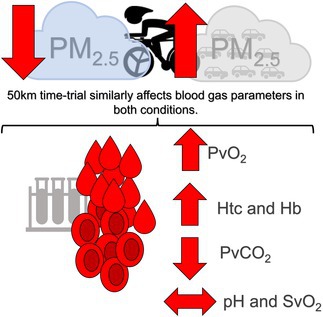

## 1 INTRODUCTION

The traffic‐related air pollution (TRAP) has emerged as an environmental risk to populations who live in low‐ and middle‐income countries (World Health Organization, 2021). TRAP is composed of a mixture of solid particles and gases (Cutrufello et al., 2011; World Health Organization, 2021), which are associated with a wide range of adverse human health effects (Brook et al., 2010). Some of TRAP constituents, such as fine particles with a diameter less than or equal to 2.5 μm (particulate matter—PM_2.5_), have been either remaining constant or increasing annually in urban centers like São Paulo, Brazil (Andrade et al., 2017). Additionally, although data about carbon monoxide (CO) have shown a reduction in this pollutant in São Paulo, higher levels of inhalation during exercise could have a deleterious effect on health and performance (Adir et al., 1999; Andrade et al., 2017; Cruz et al., 2020). Thus, residents of these areas are experiencing the harmful effects of pollution during their daily activities, emphasizing physical exercise.

During the transition from rest to exercise, there is an increase in minute ventilation (VE), resulting in an elevated airflow velocity that could increase the amount of total TRAP inhaled (Cruz et al., 2020; Rojas‐Rueda et al., 2011). Previous studies demonstrated that elevated CO inhalation might change blood gas parameters (Adir et al., 1999; Nicholson & Case, 1983; Peterson & Stewart, 1975). This could occur because carbon monoxide (CO) is a competitive inhibitor of oxygen to bind to the heme group of hemoglobin (Peterson & Stewart, 1975). Nicholson and Case (1983) analyzed the carboxyhemoglobin (COHb) levels in exercisers after outdoor running on a highway or in Central Park (NY, USA) during rush‐hour traffic and found that both conditions similarly elevated COHb levels (4%–5%) compared to pre‐exercise (Nicholson & Case, 1983). Furthermore, Adir et al. (1999) demonstrated that similar COHb levels (5.1%) post‐exercise significantly impaired exercise capacity in healthy young men. These findings suggest that by impairing oxyhemoglobin linkage, TRAP might produce a lower availability of O_2_ to the exercised muscles, which could result in impairment of exercise performance (Marr & Ely, 2010).

However, in the mentioned studies only COHb levels were analyzed; other blood gas parameters such as PvCO_2_ (partial pressure of carbon dioxide in venous blood), PvO_2_ (partial pressure of oxygen in venous blood), hematocrit (Htc), hemoglobin (Hb), and oxygen saturation in venous blood (SvO_2_) have not been measured, which may preclude new insights concerning the blood gas parameters dynamics when exercising under TRAP. In fact, there is limited information regarding the physiological outcomes of blood gas parameters during exercise under TRAP exposure (Crocker et al., 2017; Laeremans et al., 2018; Peterson & Stewart, 1975). Studies analyzing mixed venous oxygen and mixed venous oxygen saturation have shown a decrease in these variables with increasing exercise intensity in room air. Moreover, as pointed out by (Booher et al., 2003) the mixed venous content decreases as more blood returns to the heart from exercising muscles. Therefore, it is plausible to suppose that exercising under TRAP could influence blood gas parameters measured in venous blood (i.e., PvO_2_, PvCO_2_, and SvO_2_) due to increased CO inhalation.

While the data from the abovementioned studies provide relevant insights into the negative effects of CO (Adir et al., 1999; Peterson & Stewart, 1975) and TRAP (Nicholson & Case, 1983) on blood gas parameters, some methodological characteristics of these studies need to be considered. The exercise duration in these studies were typically limited to ≤30 min, while recent findings indicate that the effects of TRAP are exacerbated after 60 min of exercise (Pasqua et al., 2020). Taking these into account, experimental approaches that allow a broad analysis for a prolonged period (i.e., ≥60 min) using a real‐world and well‐controlled environment could provide new insights about the impacts of exercise performed under TRAP on blood gas parameters dynamics.

Therefore, we analyzed in the present study the effects of TRAP on blood gas parameters during prolonged exercise (i.e., 50‐km cycling time trial) in recreationally trained cyclists. Based on previous findings suggesting that pollution of the atmospheric air could impair oxygen carrying capacity during exercise (Nicholson & Case, 1983), we hypothesized that TRAP could affect blood gas parameters when compared with a filtered air condition.

## 2 MATERIALS AND METHODS

### 2.1 Participants

The sample size was estimated using G*Power software (version 3.1.9.2), utilizing an effect size of 0.36 in relation to the influence of air pollution on cycling performance (Adir et al., 1999), an alpha of 0.05, and a power of 0.95. The estimated sample size was eight participants in each group. Ten male cyclists (age: 33 ± 5 years; height: 180 ± 10 cm, and body mass: 73.4 ± 7.6 kg) volunteered to participate in this study. The cyclists had been engaged in cycling training prior to joining the study (>4 years). Mean outdoor training volume was 174 ± 89 km and 7.5 ± 3.9 h per week. Most of the training sessions were performed on a cycle path and on roads inside the campus of the University of São Paulo, where the Environmental Agency of the State of São Paulo (CETESB) has reported pollution levels that surpass the annual vehicular traffic pollutants concentration limits imposed by the WHO (i.e., PM_2.5_ >5 μg/m^3^) (World Health Organization, 2021). As inclusion criteria, participants were free from cardiovascular diseases, respiratory diseases, or recent injuries that would compromise exercise performance. They also were not taking medications or banned substances. All procedures, benefits, and risks were explained before the beginning of trials, and participants gave their written informed consent. The study was conducted according to the Declaration of Helsinki and approved by the Research Ethics Committee of School of Physical Education and Sports at University of São Paulo (process n° 3.359.652).

### 2.2 Experimental design

The participants attended the laboratory on five separate occasions, at least 48 h apart and at the same time of day (±2 h). During the first session, participants underwent anthropometric measurements (i.e., body mass and height) and performed a maximal incremental test to measure maximal oxygen uptake (VO_2_max), maximal heart rate (HRmax), and peak power output (POpeak). During the second and third visits, participants were familiarized with the 50‐km cycling TT. The fourth and fifth sessions (i.e., experimental sessions) were randomized using an online tool (http://www.jerrydallal.com/random/permute.htm) providing a counterbalanced order. Participants were oriented to maintain their training routine over the study period, but they were instructed to refrain from any exhaustive or unaccustomed exercise, alcohol, or caffeine 48 h prior to experimental sessions. All tests were performed at a controlled ambient temperature (20–24°C) and 2 h after the last meal.

### 2.3 Preliminary trial

The incremental test was performed with a 5‐min warm‐up at a power output of 100 W; then power output was increased by 30 W every 3 min. Pedal frequency was maintained between 80 and 90 revolutions per minute throughout the test. Task failure was defined as the inability to maintain the pedal frequency above 80 rpm for more than 5 s, despite strong verbal encouragement. Gas exchange was measured breath‐by‐breath throughout the trial using an automatic metabolic cart (Cortex Metalyzer 3B, Cortex Biophysik, Leipzig, Germany). The metabolic cart had been calibrated before the test using a 3‐L syringe, ambient air, and a cylinder containing a known concentration of O_2_ and CO_2_ (12% and 5%, respectively). A sensor coupled to the metabolic cart was used to measure HR. V̇O_2_max was defined as the highest V̇O_2_ computed from a 20‐s rolling average. HRmax and POpeak were defined as the highest values obtained during the test.

### 2.4 Experimental trials

Participants arrived at the laboratory and rested for 10 min in a seated position. A pre‐exercise venous blood sample (1 mL) was then collected from the antecubital vein using a blood gas syringe (safePICO self‐fill syringe, Radiometer Medical ApS, Copenhagen, Denmark). These blood samples—devoid of air bubbles and clots—were immediately analyzed for pH, PvCO_2_, PvO_2_, Hb, and SvO_2_ using an automatic blood gas analyzer (ABL800 Flex Radiometer Medical ApS, Copenhagen, Denmark).

Participants then warmed up for 5 min at 100 W with a pedal cadence of 90 rpm, followed by a self‐paced 50‐km cycling time trial (TT). Participants were instructed to complete the 50‐km TT as quickly as possible, simulating a real‐world competition. Power output (PO) was recorded at a frequency of 1 Hz via an interface connecting the cycling simulator to a computer (RacerMate One software, Seattle, Washington, USA) and averaged sustained during 50‐km TT was determined. The only visual feedback available to participants during the trial was the distance covered. Participants were instructed to maintain an upright trunk position and avoid standing while cycling. The completion time for the TT was revealed only after all trials were concluded. A second blood sample (1 mL) was collected 5 min after the end of the trial for measurement of the same variables in post‐exercise.

### 2.5 Environmental chamber

The experimental trials were performed in an environmental chamber located 20 m from the roadside, 150 m from a busy traffic crossroad in downtown São Paulo, as previously described (Pasqua et al., 2020; Silveira et al., 2022). The environmental exposure chamber was equipped with three filter systems: (I) a prefilter to collect atmospheric dust and larger particles (fine screen); (II) chemical filters, one with potassium permanganate (Purafil), to oxidize and trap gases such as formaldehyde, hydrogen sulfide, sulfur dioxide, NO, and NO_2_, and another with charcoal to trap volatile organic compounds (VOCs); (III) HEPA (High Efficiency Particulate Arrestance) to filter fine particles (PM < 0.3 mm) and biological filters to capture a wide range of gaseous pollutants (e.g., NO and NO_2_). Under the filtered air condition, the ambient air passed through these systems and was sent to the testing room via an aluminum structure (air conductor). Under the TRAP condition, the air passed through an alternate port without any filtration; therefore, individuals were exposed to a pollution mixture that corresponded to local real‐time pollution composition and levels from the nearby intersection described above. The CO concentration data were provided by CETESB pollutants monitoring station which is located approximately 100 m from the chamber. PM_2.5_ was measured during experimental conditions using an HHTP21 (Omega engineering inc. Spectris plc., Norwalk, Connecticut, USA). Temperature and relative air humidity were measured during all exposure visits using a digital thermo hygrometer (Incoterm, Porto Alegre, RS, Brazil).

### 2.6 Statistical analysis

Normality in data distribution was confirmed by Shapiro–Wilk test and reported as mean ± SD. A two‐way ANOVA (moment × condition) with repeated measures was used to compare the blood gas parameters between experimental sessions. The Cohen's *d* effect size (0.20–0.49 small, 0.50–0.79 medium, and >0.80 large) and the ETA‐squared—ŋ^2^ (0.01–0.05 small, 0.06–0.13 medium, and >0.14 large) are also reported. The significance level was set at *p* < 0.05. Statistical analyses were performed using a statistical software package (Statistica 13, StataSoft Inc., Tulsa, OK, USA).

## 3 RESULTS

### 3.1 Preliminary trial

Based on variables measured during the maximal incremental test (i.e., maximal oxygen uptake = 51.1 ± 5.1 mL·kg^−1^·min^−1^ and PO_peak_ = 311 ± 26 W), participants were classified as recreationally trained cyclists (De Pauw et al., 2013).

### 3.2 Experimental trials

**FIGURE 1:**
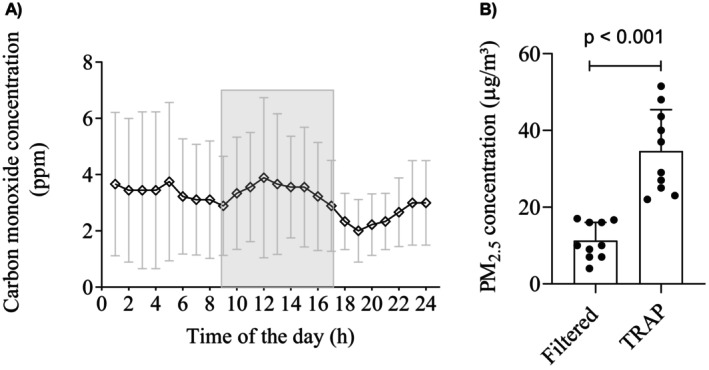
Hourly mean of ambient CO concentration in the experimental sessions and PM_2.5_ concentration measured during the 50‐km cycling time‐trial. The filled area corresponds to hours in which experimental trials were conducted. CO, carbon monoxide; PM_2.5_, particulate matter with an aerodynamic diameter lower than 2.5 μm. Data are mean ± SD. *p* < 0.05 significantly different from filtered condition.

Figure 1 (Panel a) shows the hourly mean of CO concentration measured during the 50‐km cycling time trial. The efficiency of HEPA filters was confirmed by the concentration of PM_2.5_ measured inside the environmental chamber, which was significantly lower (*t*_(9)_ = 6.22, *p* < 0.001, *d* = −1.96) in the Filtered (11.2 ± 4.7 μm/m^3^) than in the TRAP (34.6 ± 10.8 μm/m^3^) condition (Figure 1, Panel b).

**FIGURE 2:**
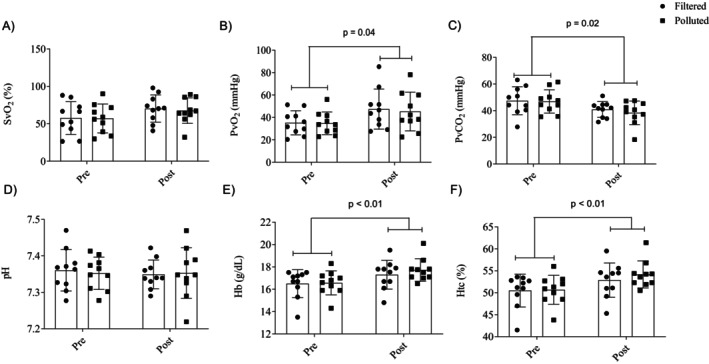
Venous blood gas parameters measured before (pre‐exercise) and after (post‐exercise) a 50‐km cycling time‐trial performed while exposed to traffic‐related air pollution (TRAP) or filtered air. Hb, hemoglobin; Htc, hematocrit; pH, potential of hydrogen; PvCO_2_, partial pressure of carbon dioxide; PvO_2_, partial pressure of oxygen; SvO_2_, oxygen saturation. Data are mean ± SD. *p* < 0.05 significantly different from pre‐exercise (*p* < 0.05).

There was no significant difference in the mean PO during the 50‐km cycling TT in filtered air (202.2 + 30.4 W) and TRAP (199.3 + 24.0 W) conditions (*t*_(9)_ = 0.12, *p* < 0.907, *d* = 0.038). There was a main effect of time for PvO_2_ (*F*_[9,81]_ = 5.8, *p* = 0.04; $\eta_{P}^{2}$ = 0.392) (Figure 2, Panel b), PvCO_2_ (*F*_[9,81]_ = 7.582, *p* = 0.02; $\eta_{P}^{2}$ = 0.457) (Figure 2, Panel c), Hb (*F*_[9,81]_ = 14.48, *p* < 0.01; $\eta_{P}^{2}$ = 0.616) (Figure 2, Panel e), and HTC (*F*_[9,81]_ = 13.87, *p* < 0.01; $\eta_{P}^{2}$ = 0.60) (Figure 2, Panel f) with PvO_2_, Hb, and HTC increasing and PvCO_2_ reducing from pre‐ to post‐trial in both conditions. There is no significant difference for SvO_2_ (*p* = 0.84). There was no main effect of condition or condition vs. moment interaction for any analyzed blood gas parameters (*p* > 0.05).

SvO_2_: oxygen saturation; PvO_2_: partial pressure of oxygen; PvCO_2_: partial pressure of carbon dioxide; pH: potential of hydrogen; Hb: hemoglobin; Htc: hematocrit. Data are mean ± SD. *Significantly different from pre‐exercise (*p* < 0.05).

## 4 DISCUSSION

In the present study, we explored whether TRAP would influence blood gas parameters during prolonged cycling exercise. Our findings indicate that the PvO_2_, Hb, HTC, pH, SvO_2_, and PvCO_2_ responses were similar between TRAP and filtered air conditions. These findings revealed the fact of endurance performance has not been impaired by TRAP could also be explained by the absence of physiological outcomes, such as blood gas parameters.

Moreover, we observed an increase in PvO_2_ and a decrease in PvCO_2_ post‐exercise on both experimental conditions. This contrasts with previous studies that reported a decreased O_2_ content in mixed venous oxygen during exercise (Casaburi et al., 1989; Lang Daum, 1983; Satoh et al., 2004). Therefore, our data suggest that venous blood might be more oxygen rich at post‐exercise than during exercise. This effect likely occurs due to higher cardiac output and higher ventilation compared with the baseline, as well as the increase of the blood flow to remove metabolites from the muscle (Korthuis, 2011). Otherwise, this increase in PvO_2_ post‐exercise could reflect a time delay in convective oxygen delivery blood flow (Korthuis, 2011; Spires et al., 2013). In addition, the increase in PvO_2_ and the decrease in PvCO_2_ could occur due to post‐exercise vasodilation, which helps maintain elevated oxygen levels in the muscles (Bangsbo & Hellsten, 1998). This, combined with reduced metabolic demand, may lead to lower oxygen extraction, resulting in increased PvO_2_ and a reduction in PvCO_2_ compared to baseline measures. This improved oxygen availability may facilitate higher venous O_2_ content even during TRAP exposure.

The increase observed in Hb and HTC post‐exercise in both conditions (i.e., TRAP and filtered air) is likely due to long‐distance exercise‐induced a hemoconcentration (Komka, Szilágyi, et al., 2022). There are some explanations for this phenomenon in the literature, such as dehydration during long‐distance exercise and the increase in metabolite levels in the blood, changing the osmotic gradient between muscle and blood (Komka, Szilagyi, et al., 2022; Komka, Szilágyi, et al., 2022). Since the PM_2.5_ can be transported by the blood (Nemmar et al., 2002), it may also contribute to a more pronounced hemodilution (i.e, plasma volume loss) due to changes in osmolarity. However, it seems that hemoconcentration is not affected by air pollution in recreationally trained cyclists.

Trained individuals who regularly perform their training routine in conditions of elevated TRAP do not seem to demonstrate a reduction in exercise performance under TRAP (Giles et al., 2018; Silveira et al., 2022). In this scenario, the methodological approach used in this study (employing an environmental chamber) offers a unique opportunity to simulate realistic scenarios under well‐controlled conditions in a real air‐polluted environments to comprehend how air pollution could affect physiological outcomes in amateur athletes who live in polluted areas. In fact, our findings showed that pollutant concentrations were consistently lower in the filtered air condition compared to the polluted condition (Figure 1, panel b). These findings suggest that the blood gas responses during prolonged endurance exercise do not appear to be affected by real‐world TRAP or to induce a reduction in exercise performance.

As mentioned above, the training status could account for some inconsistencies between physiological responses observed during exercise performed under polluted environments. There is some evidence from both animal and human studies indicating that the harmful effects of air pollution can be mitigated by prolonged periods of training in polluted environments (Silva‐Renno et al., 2018; Silveira et al., 2022). For instance, long‐term training in mice has been shown to have protective effects on various lung compartments affected by PM exposure (Silva‐Renno et al., 2018; Vieira et al., 2016). Although further research is needed to elucidate the mechanisms, it is possible that the absence of changes in blood gas parameters in the current study is due to an adaptive response to prolonged exposure to air pollutants during exercise.

The present study has some limitations that need to be considered. Firstly, blood gas parameters were determined in venous rather than arterial blood, which may be less sensitive to air pollution changes. Secondly, the relatively small sample size and the exclusion of female participants could limit the extrapolation of these findings to other populations. This is particularly relevant because previous findings have demonstrated that females would be more susceptible to the negative impacts of air pollution exposure (Marr & Ely, 2010). Lastly, in order to better understand the molecular mechanisms that may be affected by pollution in cyclists, some relevant markers could have been measured, such as 2,3‐diphosphoglycerate (Heinonen et al., 2014).

In conclusion, findings of the current study indicate that real‐world TRAP does not affect performance during a prolonged exercise and does not change physiological outcomes as venous blood gas parameters in recreational athletes who train in pollutant environments.

## FUNDING INFORMATION

This study was financed in part by the Coordenação de Aperfeiçoamento de Pessoal de Nível Superior—Brasil (CAPES)‐Finance Code 001; coordination of Superior Level Staff Improvement—Academic Excellence Program (CAPES‐PROEX). A. C. Silveira was supported by a Sao Paulo Research Foundation scholarship (FAPESP Grant: 2018/21600‐0).

## ETHICS STATEMENT

This project was approved by the Research Ethics Committee of the School of Physical Education and Sport at the University of São Paulo (Process No. 3.359.652) and was conducted in accordance with the Declaration of Helsinki. All participants provided written informed consent.

## Data Availability

The datasets generated and/or analyzed in this study are available from the corresponding author upon reasonable request.
